# Enhancing heat stress tolerance in Lanzhou lily (*Lilium davidii* var. *unicolor*) with Trichokonins isolated from *Trichoderma longibrachiatum* SMF2

**DOI:** 10.3389/fpls.2023.1182977

**Published:** 2023-06-07

**Authors:** Xing Cao, Juanjuan Sui, Haiyan Li, Wenxiu Yue, Tao Liu, Dong Hou, Jiahui Liang, Ze Wu

**Affiliations:** ^1^ Department of Environmental Art Design, College of Architecture, Yantai University, Yantai, China; ^2^ Engineering Technology Research Center of Anti-aging Chinese Herbal Medicine, Biology and Food Engineering College, Fuyang Normal University, Fuyang, China; ^3^ Vegetable Research Institute, Gansu Academy of Agricultural Sciences, Lanzhou, China; ^4^ Institute of Grassland, Flowers and Ecology, Key Laboratory of Urban Agriculture (North), Ministry of Agriculture, Beijing Academy of Agriculture and Forestry Sciences, Beijing, China; ^5^ Key Laboratory of Landscaping Agriculture, Ministry of Agriculture and Rural Affairs, College of Horticulture, Nanjing Agricultural University, Nanjing, China

**Keywords:** Lanzhou lily, Trichokonins, thermotolerance, physiological response, RNA-Seq, Hsf

## Abstract

Lanzhou lily (*Lilium davidii* var. *unicolor*) is a renowned edible crop produced in China and relatively sensitive to high temperature (HT). Trichokonins (TKs) are antimicrobial peptaibols secreted from *Trichoderma longibrachiatum* strain SMF2. Here, we report that TKs application improves the thermotolerance of Lanzhou lily. The activity of the antioxidant enzyme system (SOD, CAT, and POD), the level of heat-resistance-associated phytohormones (ABA, SA, and JA), the relative water content (RWC), the content of chlorophyll (Chl), and the net photosynthetic rate (*P*_n_) were promoted by TKs treatment in Lanzhou lily plants subjected to heat stress (HS). TKs treatment also mitigated cell injury as shown by a lower accumulation of malondialdehyde (MDA) and relative electrolyte leakage (REL) under HS conditions. RNA-seq data analysis showed that more than 4.5 times differentially expressed genes (DEGs) responded to TKs treatment under HS compared to non-HS, and TKs treatment reduced protein folding and enhanced cellular repair function under HS conditions. The analyses of DEGs involved in hormone (ABA, SA and JA) synthesis and signaling pathways suggested that TKs might improve Lanzhou lily heat tolerance by promoting ABA synthesis and signal transduction. TKs highly induced DEGs of the HSF-HSP pathway under HS, in which *HSFA2* accounted for most of the HSF family. Furthermore, TKs treatment resulted in the upregulation of heat-protective genes *LzDREB2B*, *LzHsfA2a*, *LzMBF1c*, *LzHsp90*, and *LzHsp70* involved in HSF-HSP signal pathway after long-term HS. LzHsfA2a-1 likely plays a key role in acquisition of TKs-induced thermotolerance of Lanzhou lily as evidenced by the sustained response to HS, the enhanced response to TKs treatment under long-term HS, and the high sequence similarity to LlHsfA2a which is a key regulator for the improvement of heat tolerance in *Lilium longiflorum*. Our results reveal the underlying mechanisms of TKs-mediated thermotolerance in Lanzhou lily and highlight an attractive approach to protecting crop plants from damage caused by HS in a global warming future.

## Introduction

High temperature (HT) is a major environmental stress that limits plant growth and development, particularly crop yields. As sessile organisms, plants have evolved various physiological and molecular defense mechanisms in response to heat stress (HS). HS causes the overproduction of reactive oxygen species (ROS), lipid peroxidation, photoinhibition, protein denaturation and degradation, and RNA damage, which are responsible for an imbalance in cellular homeostasis (Bokszczanin and Fragkostefanakis, 2013). Meanwhile, factors involved in signaling cascades and transcriptional control, such as ion transporters, proteins, osmoprotectants and antioxidants, are activated by HS to maintain cellular homeostasis (Hasanuzzaman et al., 2013). Understanding the mechanisms underlying heat stress response (HSR) are essential to improving thermotolerance *via* conventional breeding, genetic engineering or protectants applications. The unfolded protein response in endoplasmic reticulum and the heat shock response in cytoplasmic are two evolutionarily conserved systems that protect plants from HS (Li et al., 2020). A complex signal transduction network may integrate signals from all these different sensors involving calcium fluxes, ROS, calmodulin, CDPKs, MAPKs, phosphatases, and transcriptional regulators such as heat stress transcription factor (HSF), MBF1c, WRKY, NAC, DREB, bZIP, MYB, and bHLH (Koini et al., 2009; Zhao et al., 2017; Li et al., 2020; Cai et al., 2021; Haider et al., 2021; Saini et al., 2022; Tian et al., 2022). Among the complex HS signal transduction networks, heat stress transcription factor- heat shock proteins (HSF-HSP) pathway plays a central role in the acquisition of heat tolerance in plants. HS leads to the synthesis and accumulation of HSPs functioning as molecular chaperones to control protein homeostasis. *HSP* genes are regulated by HSFs that can recognize and bind to heat shock elements (HSEs) in the *HSP* promoter regions. It is reported that HSFA2 is a master transcriptional regulator of thermomemory in *Arabidopsis* (Liu et al., 2019; Friedrich et al., 2021).

Lanzhou lily (*Lilium davidii* var. *unicolor*), an endemic species in China, is a famous edible crop for its large, white, sweet bulb scales (Cao et al., 2020). Because of its high edible, medicinal, and ornamental value, Lanzhou lily plays important roles in landscape agriculture and rural revitalization. Lanzhou lily well adapts to cool and humid conditions, and HT may lead to growth stagnation and bulb degradation. Due to its poor heat resistance, HT is a key limiting factor to the annual production of Lanzhou lily bulbs. Measures such as breeding or plant growth regulator (PGR) application must be taken to overcome this limitation. In some studies, OT lily cultivar ‘Jinmen’ selected from the cross-combination ‘T11’(♀)and ‘D74’(♂) shows strong heat resistance (Cui et al., 2014). CaM3, HSFA1, HSFA2, HSFA3, and DREB2 are important components of HS signal transduction in lilies (Xin et al., 2010; Cao et al., 2013; Gong et al., 2014; Xin et al., 2017; Wu et al., 2018a; Wu et al., 2018b). Pretreatment of salicylic acid (SA) increases the heat tolerance of lily by enhancing the activities of antioxidant systems (Chen et al., 2008). To date, protectants for improving the thermotolerance of Lanzhou lily have not been reported.

In recent times, exogenous applications of protectants in the form of osmoprotectants (e.g., proline, Pro; glycine betaine, GB; and trehalose, Tre) (Wahid and Shabbir, 2005; Kaushal et al., 2011; Luo et al., 2014), phytohormones (e.g., abscisic acid, ABA; jasmonic acid, JA; epibrassinolide, EBL; and salicylic acid, SA) (Wang and Li, 2006; Scalabrin et al., 2016; Alam et al., 2018; Islam et al., 2018), signaling molecules (e.g., nitric oxide, NO) (Li X, et al., 2014), polyamines (e.g., putrescine, Put; spermidine, Spd; and spermine, Spm) (Fuller and Gerner, 1982; Kumar et al., 2012), trace elements (e.g., selenium, Se; and silicon, Si) (Malerba and Cerana, 2018; Hussain et al., 2019) and nutrients (e.g., nitrogen, N; phosphorus, P; potassium, K, and calcium, Ca) (Waraich et al., 2012; Cao et al., 2013) have been found effective in mitigating HT stress-induced damage in plants. Some of the protectants, also known as plant inducers, can be synthetic compounds, natural products or living microorganisms. When applied exogenously at low doses before upcoming HS or at the early stage of stress occurrence, inducers can rapidly activate the plant’s defense systems and then enhance plant resistance to HS. Beneficial microbes, such as plant growth-promoting bacteria and plant growth-promoting fungi, can modulate the plant transcriptome and metabolome to induce thermotolerance (Shekhawat et al., 2022). Compounds produced by *Enterobacter* sp. SA187 trigger ethylene signaling in *Arabidopsis* to regulate HS-tolerance *via* increased expression of *EIN3* and *HSFA2* genes (Zélicourt et al., 2018). 2-Amino-3-methylhexanoic acid (AMHA) isolated from *Alternaria alternata* facilitates HT resistance by alleviating physiological damage in field-grown tea plants due to improved photosynthetic performance, osmotic adjustments and antioxidant enzyme activities (Yang et al., 2023). Trichokonins (TKs) are antimicrobial peptaibols extracted from *Trichoderma longibrachiatum* strain SMF2 (Song et al., 2006). Our previous studies revealed that TKs exhibited broad-spectrum antimicrobial activity against bacteria, fungal phytopathogens and TMV (Luo et al., 2010; Shi et al., 2012; Li H, et al., 2014). However, to our knowledge, no studies have addressed the function of TKs on plant abiotic stresses. Here, we demonstrate the effect and mechanism of TKs on regulating the thermotolerance of Lanzhou lily.

## Materials and methods

### Plant materials and growth conditions

Lanzhou lily (*Lilium davidii* var. *unicolor*) bulbs were derived from Qilihe district, Lanzhou city, China, and cultivated in Dongchangfu district, Liaocheng city (S 115°97’, W 36°45’), China. Sterilized bulbs with uniform size (bulb circumference 5.0 ± 0.15 cm) were potted in plastic pots (height: 9 cm, top square with side length: 10 cm, bottom square with side length: 8 cm) containing a sterile 1:1 mixture of sphagnum peat and sand and grown in a greenhouse under suitable growth conditions (16 h/8 h light/dark and 25°C/20°C light/dark). After cultivation for 21 d, healthy uniform size plants with 9.40 ± 0.20 cm high and 0.34 ± 0.02 cm stem diameter were prepared for thermotolerance test. After cultivation for 28 d, healthy uniform size plants with 10.61 ± 0.35 cm high and 0.43 ± 0.03 cm stem diameter were prepared for physiology and RNA-seq experiments.

### Trichokonins preparation and heat treatment

Trichokonins (TKs) stock solution with a 5 mg/mL concentration prepared as previously described (Luo et al., 2010). In different experiments, distilled water was used for further dilution of TKs. Roots of Lanzhou lily plants were treated with 30 mL solution of 0.5, 1, 2, 4, and 8 mg/L TKs, respectively, at 22°C for 12 h, then subjected to a direct HS at 40°C for 72 h. Control plants were treated with distilled water. After HS, the survival rate was recorded with a 30-d-recovery period. Each treatment consisted of three replicates and twelve individual plants served as one replicate.

### Physiological measurements

After root-irrigation with 30 mL solution of 2 mg/L TKs or 30 mL distilled water (non-treated control) at 22°C for 12 h, Lanzhou lily plants were exposed to different durations (24, 48, and 72 h) at 40°C (HS conditions) or 22°C (non-stress control). The middle part leaves of plants were collected for physiological indices measurement. Five plants were pooled together as one biological replicate, and each treatment was repeated three times.

The relative water content (RWC) was calculated as previously described (Lafitte, 2002). Relative electrolyte leakage (REL) was evaluated according to the method of Lutts et al. (1996). Chlorophyll (Chl) content was quantified following the method of Frank et al. (2005) with slight modification. Net photosynthetic rate (*P*_n_) was measured as described by Zhang et al. (2013) using the portable photosynthesis measurement system LI-6800 (LI-COR, USA). The malondialdehyde (MDA) content and the activities of superoxide dismutase (SOD, EC 1.15.1.1), catalase (CAT, EC 1.11.1.6), and peroxidase (POD, EC 1.11.1.7) were assayed using MDA Test Kit (A003-1), Total SOD Assay Kit (A001-1), CAT Assay Kit (A007-1), and Plant POD Assay Kit (A084-3), respectively, according to the mentioned manufacturer’s protocol (Nanjing Jiancheng, Nanjing, China).

The ABA, SA, and JA were extracted and measured as described previously, with minor modifications (Liu et al., 2014). Briefly, about 500 mg of plant leaf tissue was ground into a fine powder in liquid nitrogen and then phytohormones were extracted twice with acetonitrile and purified with a Poroshell 120 SB-C18 column. The quantification of ABA, SA, and JA was determined by high-performance liquid chromatography-electrospray ionization-tandem mass spectrometry (HPLC-ESI-MS/MS) using an Agilent 1260 HPLC coupled to an 6420A MS (Agilent Technologies Inc., USA).

### *De Novo* transcriptome sequencing and analysis

Two groups of Lanzhou lily plants were prepared for RNA-seq. One group was the Lanzhou lily plants treated with 2 mg/L TKs or distilled water for 12 h at 22°C (non-HS, RT), marked as “TKs” and “W” respectively. The other group of samples was Lanzhou lily plants treated with 2 mg/L TKs or distilled water for 12 h at RT followed by 40°C HS treatment for 12 h, marked as “HS+TKs” and “HS” respectively. The middle part leaves of these Lanzhou lily plants were immediately collected and quickly put into liquid nitrogen for RNA sequencing. The total RNA of the leaf samples was extracted using a CTAB-PBIOZOL reagent (Bioflux, Hangzhou, China). RNA integrity, purity and concentration were evaluated using 1% agarose gel electrophoresis and NanoDrop 2000 (Thermo Fisher Scientific Inc., USA), and Agilent 2100 (Agilent Technologies Inc., USA). The transcriptome analysis was performed in BGI (Shenzhen, China). The mRNA libraries were constructed according to Illumina standard instructions and subsequently sequenced on a BGISEQ-500 platform, which generated raw data of 150-bp paired-end (PE150) reads. Then, *de novo* assembly based on the clean data was performed using the Trinity program. All unigene sequences were aligned to Nt nucleotide databases using Blastn. The NCBI non-redundant (Nr) database, the Swiss-Prot protein database, the eukaryotic orthologous groups/clusters of orthologous groups of proteins (KOG/COG) database, the gene ontology (GO) database, and the Kyoto encyclopedia of genes and genomes (KEGG) database were aligned by Blastx. Each unigene was functionally annotated based on the protein sharing the highest sequence similarity with the given unigene. Fragments per kilobase of the transcript per million mapped reads (FPKM) were used to estimate the quantification of gene expression levels. A corrected p-value of <0.05 and |log2foldchange| ≥1 was set as the threshold for significant differential expression. All sequencing reads were entered into the NCBI, and the sequencing data are available at accession number PRJNA934416. KEGG analysis and GO analysis using the Omic Share platform (https://www.omicshare.com/). TBtools (https://github.com/CJ-Chen/TBtools/releases) and Graphpad prism 9 (https://www.graphpad.com/) were used to make heat maps and pie charts respectively.

### Genes expression analysis by qRT-PCR

Lanzhou lily plants were treated with 30 mL solution of 2 mg/L TKs or 30 mL distilled water at 22°C for 12 h, then subjected to a direct HS at 40°C at intervals of 0, 3, and 12 h. The middle parts of the leaves of the plantlets were collected and immediately frozen in liquid N_2_ for RNA extraction. Total RNA was extracted from 100 mg of Lanzhou lily leaves with an RNAprep Pure Plant Kit (Tiangen, China). First-strand cDNA was synthesized using HiScript III 1st Strand cDNA Synthesis Kit (+gDNA wiper) (Vazyme, China). The expression levels of *LzDREB2B*, *LzHsfA2a*, *LzMBF1c*, *LzHsp90*, and *LzHsp70* genes in Lanzhou lily were determined by real-time quantitative RT-PCR (qRT-PCR). qRT-PCR was performed using the SYBR Green Supermix (Takara) on a Roche LightCycler 480 II (Roche, Switzerland). Gene expression data was normalized using *18S* rRNA as internal control. Primers designed for the qRT-PCR analysis are listed in **Table 1**.

**Table 1:** Primers sequences used for qRT-PCR assays.

Gene	Primer sequence (5′-3′)
*LzDREB2B*	F: CACTGCTGTGTTGGTATTCCAGACT
	R: CACTAGCAGCATACAAGCCTAATCCT
*LzHsfA2a*	F: CAGACTGAGGTCGAGTTGGAAG
	R: AACACAGCCCTCTTATCTTCTCG
*LzMBF1c*	F: AATCAGGCGGTGCTTGCGAAGAT
	R: CGAAGACAATGAACTAAAGTCCATCC
*LzHsp90*	F: CTGAGGTACTTGGGAAGAAGTTCATC
	R: GTGATGTTGAGTTCAAGGCTCTACTG
*LzHsp70*	F: CCAGAGGCAGATTATGAAGAGGTC
	R: GGCTTAACAGTCCTCACTAAGGGTAG
*18S rRNA*	F: AGTTGGTGGAGCGATTTGTCT
	R: CCTGTTATTGCCTCAAACTTCC

Forward (F) primer / Reverse (R).

### Cloning and sequence analysis of LzHsfA2a

Total RNA isolation and first-strand cDNA synthesis were performed as described above. The specific forward primer SPF1 (5′-ATGGCAAGTGAGATGACGAAG-3′) and specific reverse primer SPR1 (5′-TTAAGTCTGGGAATCAAGATAACC-3′) were designed to amplify the coding sequence of *LzHsfA2a.* The PCR procedure was as follows:1 cycle of 5 min at 95°C; 35 cycles of 30 s at 95°C, 30 s at 54°C and 1 min 30 s at 72°C; and finally 1 cycle of 10 min at 72°C. The coding sequence of *LzHsfA2a* was obtained by sequencing and then translated into amino acids using ExPASy (http://web.expasy.org/translate/).The protein amino acid sequence was analyzed using the ExPASy Proteomics Tools online (http://au.expasy.org/tools/). Amino acid multiple alignments were performed using DNAMAN5.0 software, and a phylogenetic tree was constructed by the neighbor-joining method using MEGA7.0 software.

### Statistical analysis

Data are the average of three independent experiments and shown as mean ± SD. The statistical analysis was performed by One-way ANOVA and Duncan’s multiple range test using SPSS 18.0 software. A p-value < 0.05 was considered statistically significant.

## Results

### TKs treatment rescued Lanzhou lily seedlings from death under HS

**Figure 1:**
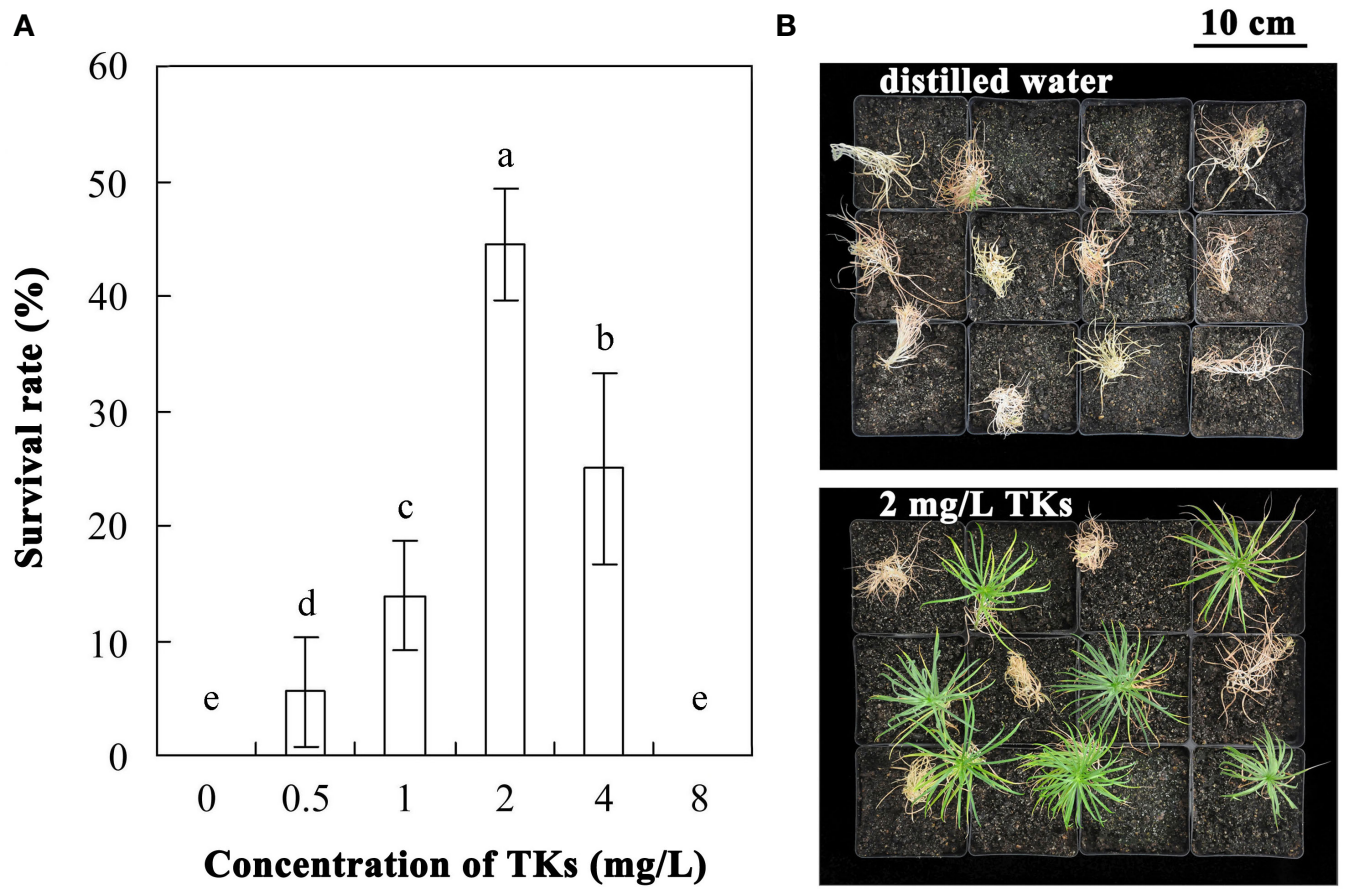
Effects of TKs on the thermotolerance of Lanzhou lily plants. **(A)** The survival rate of Lanzhou lily plants treated with different concentrations of TKs (0.5, 1, 2, 4, or 8 mg/L) or distilled water (control) after HS at 40°C for 72 h followed by a 30-d-recovery period. **(B)** Phenotype of Lanzhou lily plants treated with distilled water (upper) or 2 mg/L TKs (lower) with a 30-d-recovery period after HS at 40°C for 72 h. Each treatment included twelve plants. Data are means ± SD of three biological replications. Different letters indicate significant differences at *P <*0.05 (Duncan test).

The survival rate of Lanzhou lily plants treated with different concentrations of TKs or distilled water was assayed with a 30-d-recovery period after HS at 40°C for 72 h (**Figures 1A, B** **)**. Compared to the control, application of TKs improved the survival rate. Treatment with 0.5 or 1 mg/L TKs resulted in increased survival rate, and the effect of 2 or 4 mg/L TKs was more significant, while the survival rate decreased sharply with 8 mg/L TKs. The survival rate of Lanzhou lily plantlets treated with 2 mg/L TKs was 44.4 percentage points higher than that of the control. According to the results, treatment of 2 mg/L TKs was used for the subsequent physiology and molecular biology experiments.

### TKs treatment mitigated membrane injury and photosynthetic damage caused by HS

**Figure 2:**
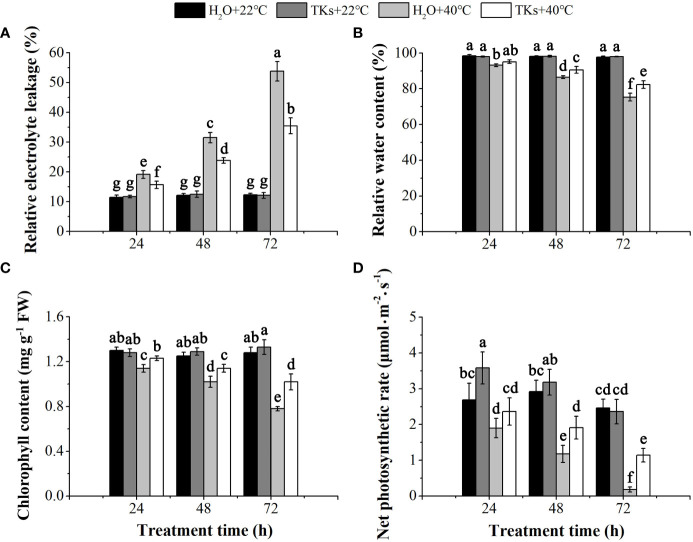
Effects of TKs on REL, RWC, *P*n, and Chl content of Lanzhou lily plants under HS conditions (24, 48, and 72 h at 40°C) and non-stress conditions (24, 48, and 72 h at 22°C). **(A)** Relative electrolyte leakage (REL); **(B)** Relative water content (RWC); **(C)** Chlorophyll (Chl) content; **(D)** Net photosynthetic rate (*P*n). Different lowercase letters indicate significant differences at P <0.05 (Duncan test).

REL is the efficient indicator of cell membrane stability. TKs treatment had little effect on the changes in REL compared with non-treated control at 22°C (p>0.05) (**Figure 2A**). REL increased significantly with time during HS, indicating heat stress damaged the membrane system and caused the leakage of electrolytes from cells. After a 72-h heat treatment, a 2.93-fold increase in REL was found in the presence of 2 mg/L TKs, significantly lower than the corresponding value of the non-treated control (4.38-fold). These results showed that exogenous TKs significantly alleviated the electrolyte leakage caused by HS.

HS brings about a water deficit in the plant, and plant water status can be measured as leaf RWC. TKs and non-TKs treated plants maintained similar levels of leaf RWC under the non-stress conditions (**Figure 2B**). Leaf RWC of both non-TKs and TKs treated plants decreased during prolonged periods of HS compared to the non-stress control, while TKs treatment resulted in greater leaf RWC compared to non-TKs treated plants. After 72 h of HS, the leaf RWC of TKs-treated plants was 7.04 percentage points significantly higher than that of the non-TKs treated plants. The findings suggested that TKs treatment gave rise to retardation in leaf dehydration under HS.

The photosynthetic capacity of plants was positively correlated with chlorophyll content. Under the non-stress control conditions, TKs application had no significant effect on chlorophyll content (**Figure 2C**). HS led to decrease in chlorophyll content, especially in the later stage (72 h). However, the chlorophyll content was significantly higher in TKs-treated seedlings than in the non-treated control. *P*_n_ showed the same tendency with chlorophyll content during the HT period (**Figure 2D**). *P*_n_ of the TKs-treated plants decreased by 51.69% at 72 h with HS, significantly lower than the corresponding value (92.68%) of plants without TKs. These results indicated that applying TKs might compensate for the photosynthetic damages resulting from HS in Lanzhou lily.

### TKs treatment enhanced ROS-scavenging ability under HS

**Figure 3:**
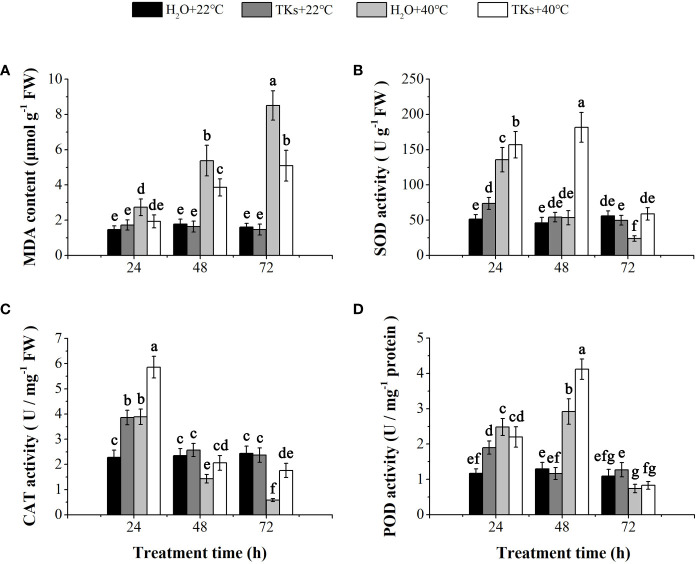
Effects of TKs on MDA content, and SOD, CAT, and POD activities of Lanzhou lily plants under HS conditions (24, 48, and 72 h at 40°C) and non-HS conditions (24, 48, and 72 h at 22°C). **(A)** MDA content; **(B)** SOD activity; **(C)** CAT activity; **(D)** POD activity. Different lowercase letters indicate significant differences at P <0.05 (Duncan test).

HS causes overproduction of ROS resulting in oxidative stress. The end products of lipid peroxidation due to heat-induced oxidative stress are reactive aldehydes, one of which is MDA. No significant differences in MDA content were observed between the TKs-treated plantlets and non-treated control under normal temperature (**Figure 3A**). In comparison with the non-stress control, long-term HS (48-72 h) significantly increased MDA content in Lanzhou lily plants with or without TKs. MDA content of plants with TKs treatment increased by 2.46-fold after HS, significantly lower than that in no-treated control (increased by 4.32-fold).

Correspondingly, plants have evolved a protective enzymatic system which consists of SOD, CAT, POD, etc., to mitigate and repair the damage initiated by ROS. TKs application increased the activities of SOD, CAT, and POD in the early stage (24 h) under non-HS conditions. Under HS conditions, treatment with TKs significantly enhanced SOD and CAT activities within 72 h, and POD activity at 48 h (**Figures 3B–D**). These results indicated that TKs treatment improved antioxidant enzyme (SOD, CAT, and POD) activities under HT conditions, which might be responsible for the decreased MDA content.

### TKs treatment increased heat-resistance-associated hormones level under HS

**Figure 4:**
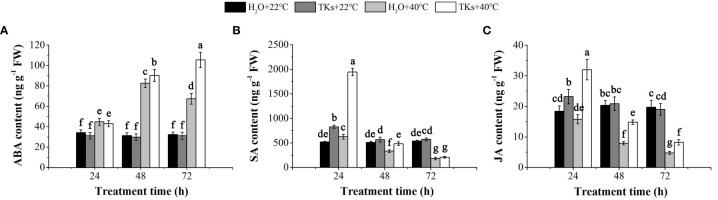
Effects of TKs on ABA **(A)**, SA **(B)** and JA **(C)** content of Lanzhou lily plants under HS conditions (24, 48, and 72 h at 40°C) and non-HS conditions (24, 48, and 72 h at 22°C). Different lowercase letters indicate significant differences at P <0.05 (Duncan test).

Phytohormones, such as ABA, JA, SA, Gibberellin (GA), and Brassinosteroids (BRs), are well-known plant growth regulators that mediate adaptations to HT conditions (Waadt et al., 2022). In this study, HPLC-ESI-MS/MS assays were conducted to detect the change of ABA, SA, and JA content in Lanzhou lily plants treated with TKs under non-HS and HS conditions. While TKs application did not significantly changed the ABA content in the absence of HS, the HT-induced ABA level was further promoted by TKs treatment (**Figure 4A**). Under stress-free conditions, SA and JA content increased by 59.29% and 26.03%, respectively, at 24 h by the addition of TKs (**Figures 4B, C** **)**. Compared to the non-treated control, Lanzhou lily plants supplement with TKs maintained a higher SA content during 48 h HS and JA content during 72 h HS. These results demonstrated that TKs treatment promoted the level of heat-resistance-associated phytohormones (ABA, SA, and JA) in Lanzhou lily plants exposed to HS.

### TKs treatment reduced protein folding and enhanced cellular repair function under HS

Transcriptome sequencing and analysis of Lanzhou lily were performed to investigate the gene activity treated with TKs under non-HS and HS. A total of 12 cDNA libraries were prepared, and 62.9 Gb clean reads were obtained. An average of 95.59% of the sequence bases had quality scores (Q-scores) of Q20% or higher (**Supplementary Table 1**). To evaluate the completeness of transcriptome libraries and annotations of all the assembled unigenes, the annotated sequences corresponded to the known nucleotide sequences of plant species, with 25.61%, 19.46%, and 6.64% matching with *Elaeis guineensis*, *Phoenix dactylifera*, and *Ananas comosus*, respectively, and the remaining 48.29% matching with others (except for the top three matched species) (**Supplementary Figure 1A**). Through the BLASTx alignment against the public protein databases including Nt, SwissProt, KOG, KEGG, and Nr, a total of 40,466 unigenes were annotated and matched to all of the public protein databases (**Supplementary Figure 1B**).

By analyzing W vs. TKs under non-HS, 7266 differentially expressed genes (DEGs) [|Log2 fold-change| (|Log2 FC|) ≥ 1, *p*-value < 0.05] were identified by comparison, of which 3865 DEGs were down-regulated by TKs treatment, while 3401 DEGs were up-regulated (**Supplementary Figure 2**). During the TKs treatment and non-TKs treatment under HS, 33,387 DEGs were identified that was more than 4.5 times the number of DEGs under non-HS. Of these, 13,097 DEGs were revised downwards, while 20,290 DEGs were revised upwards (**Supplementary Figure 3**).

**Figure 5:**
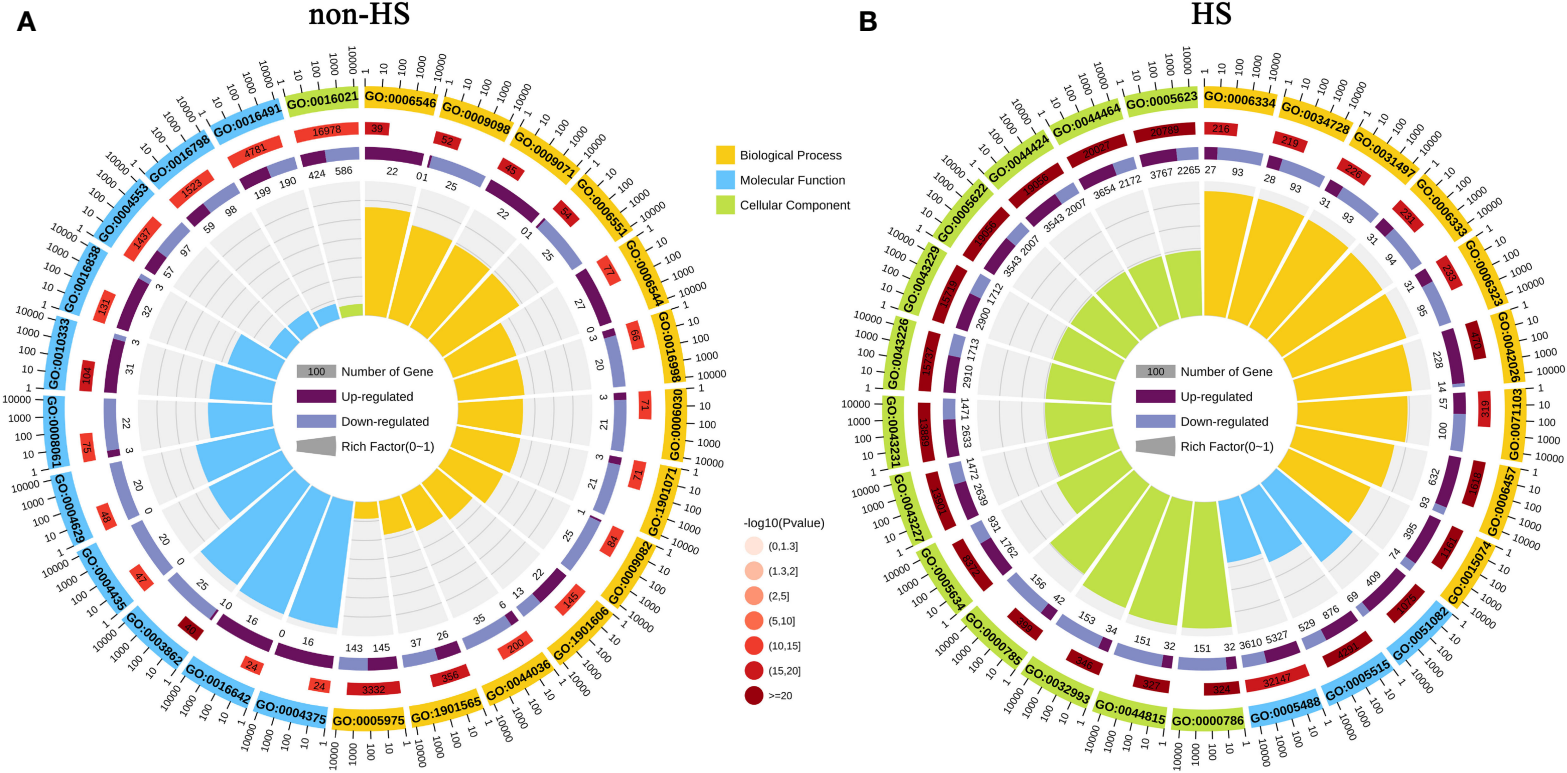
GO enrichment analysis of DEGs in response to TKs treatment under non-HS conditions **(A)** and HS conditions **(B)**.

GO enrichment analysis and KEGG enrichment analysis were performed to explore the functions of DEGs responding to TKs treatment under non-HS and HS. The top 25 GO enrichment terms are shown in **Figure 5**. Under the non-HS conditions, the functions of DEGs were mainly concentrated in the “carbohydrate metabolic process (GO:0005975)” and “organonitrogen compound catabolic process (GO:1901565)” of Biological Process, “oxidoreductase activity (GO:0016491)”, “hydrolase activity, acting on glycosyl bonds (GO:0016798)”, “hydrolase activity, hydrolyzing O-glycosyl compounds (GO:0004553)” of Molecular Function, and “integral component of membrane (GO: 0016021)” of Cellular Component (**Figure 5A**). Compared with the GO enrichment of DEGs at non-HS, the GO enrichment of DEGs of HS vs. HS+TKs had significant changes. First, the proportion of GO_Level of “Cellular Component” had increased in the top 25 GO enrichment, especially in the “Cell (GO:0005623)” and “intracellular (GO:0005622)” of the Cellular Component. Second, in the GO_Level of Biological Process and Molecular Function, “DNA integration (GO:0015074)” and “protein folding (GO:0006457)”, “protein binding (GO:0005515)” and “unfolded protein binding (GO:0051082)” showed higher p-value, respectively (**Figure 5B**).

**Figure 6:**
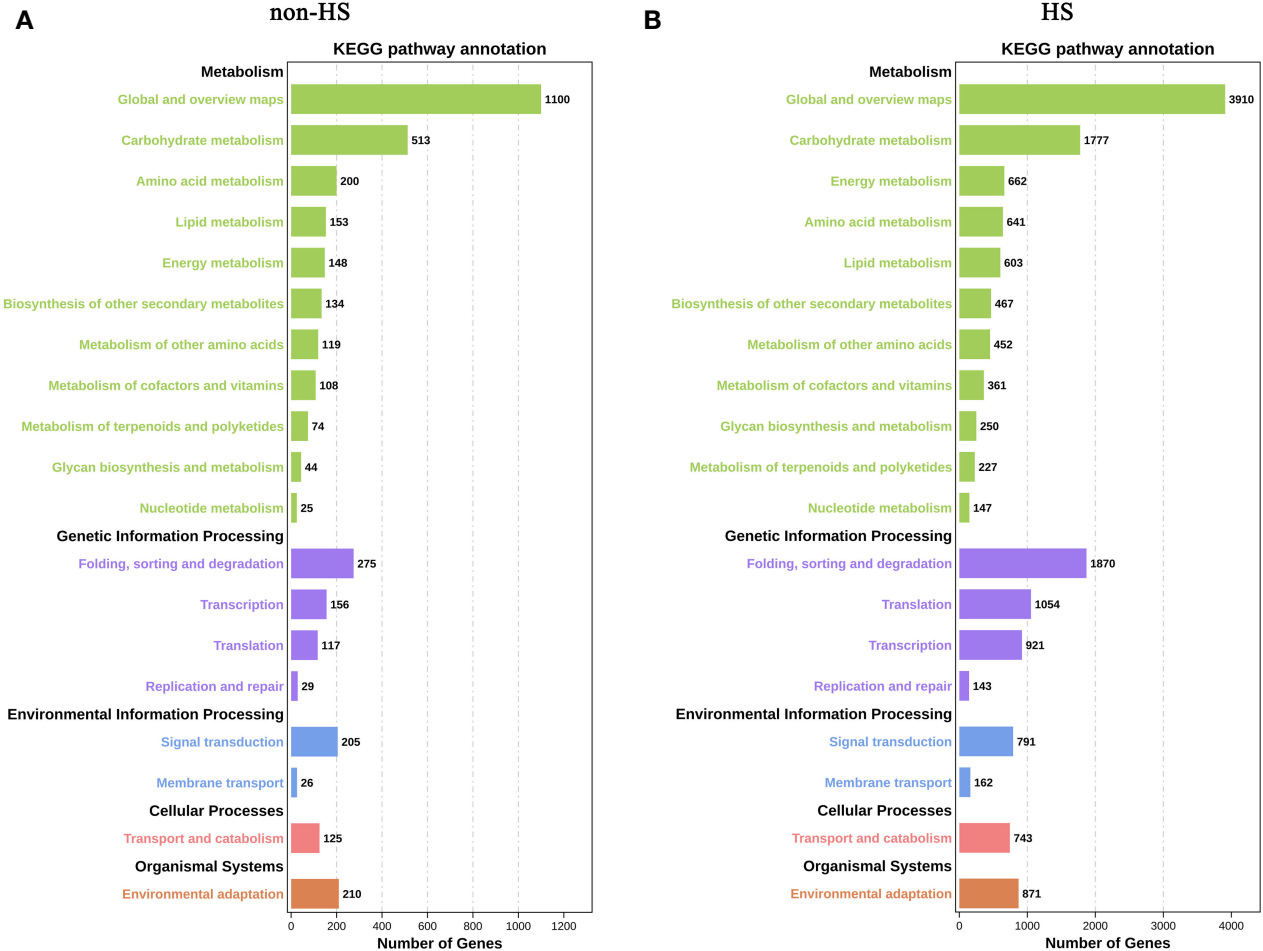
KEGG enrichment analysis of DEGs in response to TKs treatment under non-HS conditions **(A)** and HS conditions **(B)**.

In KEGG enrichment analysis under non-HS, the following terms were most enriched: “Global and overview maps” and “Carbohydrate metabolism” of Metabolism; “Folding, sorting and degradation” of Genetic Information Processing; “Signal transduction” of Environmental Information Processing; and “Environmental adaptation” of Organismal Systems (**Figure 6A**). Compared with KEGG enrichment of W vs. TKs under non-HS conditions, KEGG enrichment under HS did not change significantly, except for the relative increase in the proportion of “Energy metabolism” of Metabolism and “Translation” of Genetic Information Processing in the top 20 KEGG enrichment (**Figure 6B**).

These results suggested that TKs treatment at non-HS conditions may mainly affect the carbohydrate metabolism of Lanzhou lily plants, while TKs treatment under HS may improve heat tolerance by reducing protein folding and enhancing cellular repair function.

### TKs treatment affected the synthesis and signal transduction of ABA, JA and SA under HS

**Figure 7:**
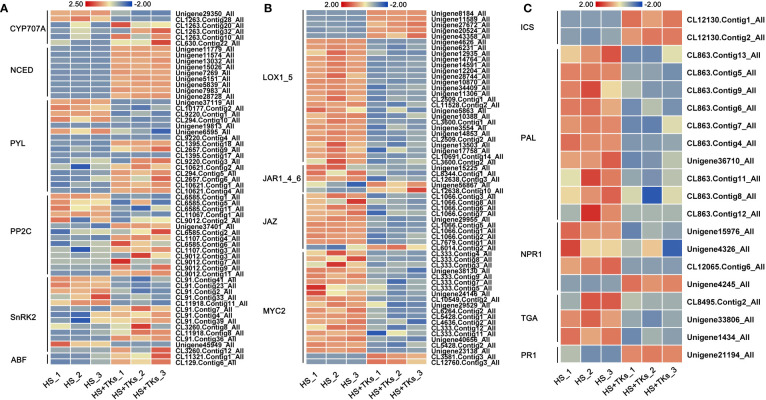
The heat map of DEGs involved in hormone synthesis and signaling pathways. Heatmaps showing the relative expression of DEGs involved in the synthesis and signaling pathways of ABA **(A)**, JA **(B)**, and SA **(C)**.

Hormone pathway is considered to be one of the main pathways of plant HS signal transduction. Based on our previous results (**Figures 4** **-** **6**), we further focused on the activity of key genes in the synthesis pathway and signaling pathway of ABA, JA, and SA. Compared to HS treatment, the genes encoding the key enzyme for ABA synthesis—*NCEDs* (*9-cis-epoxycarotenoid dioxygenases*) were significantly up-regulated by HS+TKs treatment, and the key ABA catabolism enzyme encoding genes—*CYP707As* were partially down-regulated (**Figure 7A**), indicating that TKs treatment could promote ABA synthesis under HS, which was consistent with the previous results (**Figure 4A**). The expression trends of three key genes, *PYL* (*pyrabactin resistance 1-like*), *PP2C* (*type 2C protein phosphatases*), and *SnRK2* (*SNF1-related protein kinases 2*) in the ABA signaling pathway were inconsistent, but the expression of all *ABFs*, the downstream genes of ABA signaling pathway, were up-regulated, indicating that TKs treatment might activate the signal transduction of ABA under HS.

*LOXs* (*lipoxygenases*) are genes encoding the key enzyme in the JA synthesis pathway. Among 27 differentially-expressed-*LOXs* under HS+TKs treatment compared to HS treatment, five were up-regulated (**Figure 7B**). As genes encoding key enzymes in the isochorismate synthase pathway of SA biosynthesis, the expression of *ICSs* (*isochorismate synthase*) were all significantly up-regulated, but the expression of *PALs* (*phenylalanine ammonia-lyase*), genes encoding key enzymes in the phenylalanine ammonia-lyase pathway of SA biosynthesis, were significantly down-regulated by HS+TKs treatment (**Figure 7C**). The up-regulation of *ICS* and *LOX* genes under HS+TKs treatment compared to HS treatment may be partly responsible for the increase in SA and JA levels (**Figures 4B, C** **)**. In addition, we found that the expression of key genes in the JA signaling pathway (*JAR*, *JAZ*, and *MYC2*) and SA signaling pathway (*NPR1* and *TGA*) was consistently down-regulated at HS vs. HS+TKs, but the expression of *PR1* (*pathogenesis-related gene 1*), the gene downstream in the SA signaling pathway and involved in plant disease resistance, was significantly up-regulated.

These results suggested that TKs treatment had effects on the synthesis and signal transduction of these above phytohormones under HS, and TKs could improve the heat tolerance of Lanzhou lily mainly by promoting ABA synthesis and signal transduction.

### TKs treatment had a significant impact on HSF-HSP pathway under HS

**Figure 8:**
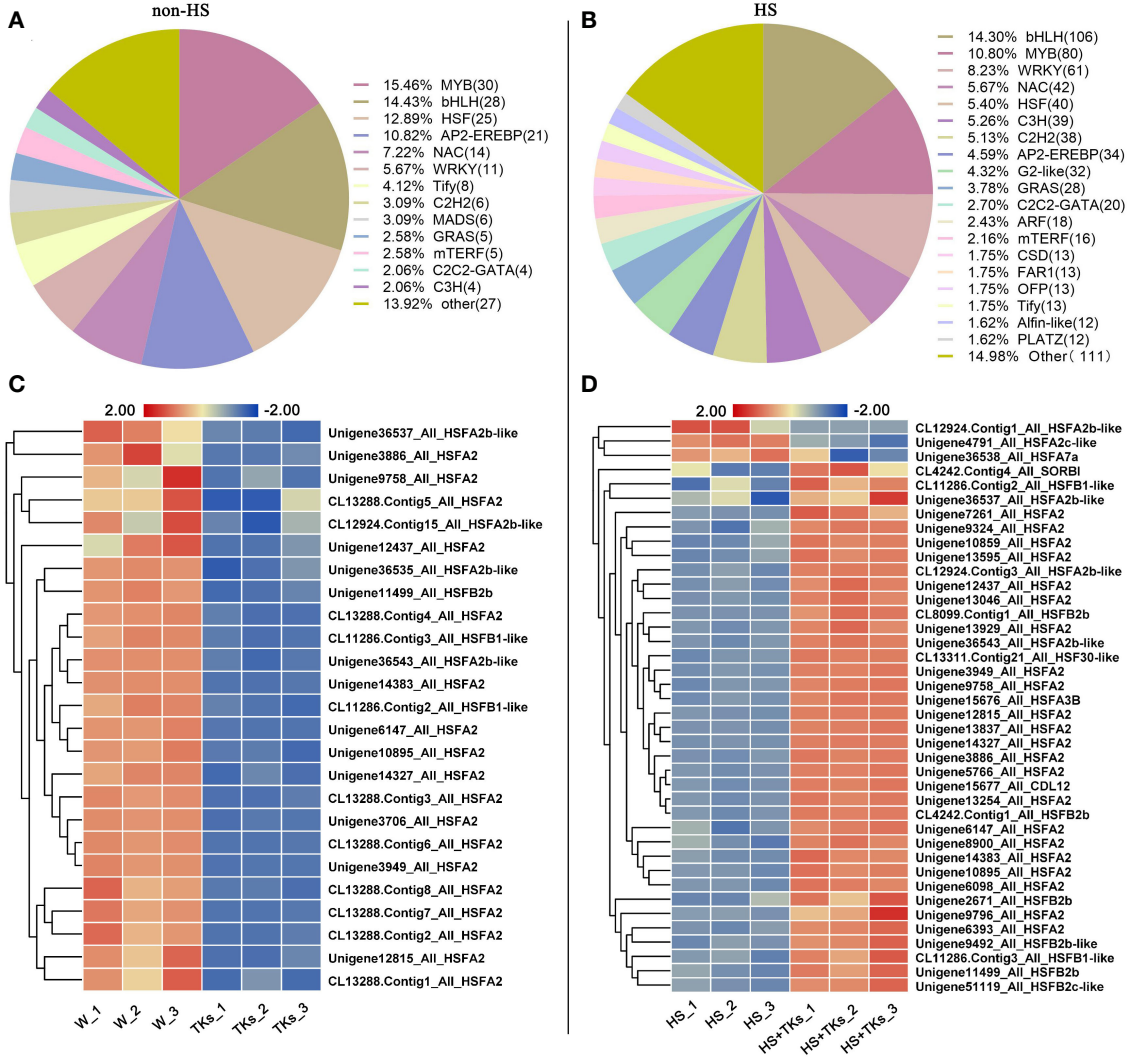
Analysis of differentially expressed transcription factors and differentially expressed *HSF* members. **(A)** Transcription factor ratio of W vs. TKs under non-HS conditions. **(B)** Transcription factor ratio of HS vs. HS+TKs. **(C, D)** The heat map of differentially-expressed-*HSFs* responding to TKs treatment at non-HS **(C)** and HS conditions **(D)**.

Transcription factors (TFs) are important proteins that regulate gene expression and play critical roles in plant abiotic stress responses. Among the DEGs, 194 annotated TFs responding to the TKs treatment under non-HS were identified, among which the MYB family, bHLH family, HSF family, AP2-EREBP family, NAC family and WRKY family accounted for 15.46%, 14.43%, 12.88%, 10.82%, 7.22%, and 5.67%, respectively (**Figure 8A**). Of the 92 annotated-*HSFs* in transcriptome data, 25 differentially-expressed-*HSFs* in response to TKs treatment were all down-regulated (**Figure 8C**). It was shown that TKs might act as a suppressor for *HSFs* under non-HS conditions.

**Figure 9:**
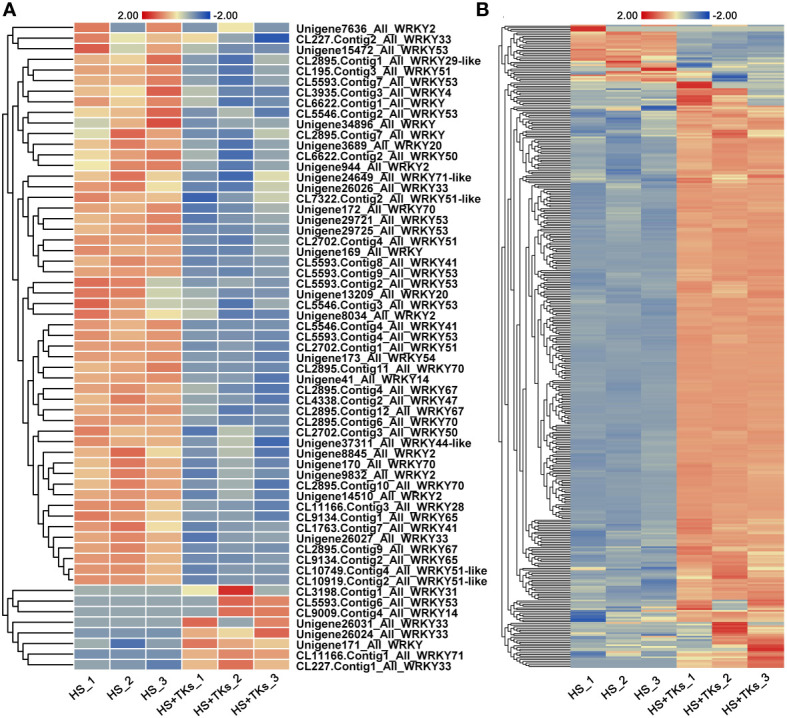
The heat map of differentially-expressed-*WRKYs* **(A)** and *HSP* family genes **(B)** responding to TKs treatment under HS conditions. The gene_ID in **(B)** was shown in **Supplementary Table 3**.

We further explored TFs in response to TKs treatment at HS. Among the 33,387 DEGs, 741 differentially-expressed-TFs were identified, mainly distributed in the families of bHLH, MYB, WRKY, NAC, HSF and C3H. Several WRKYs and HSFs were identified as crucial positive TFs in HSR of ornamental lily based on our earlier investigations (Wu et al., 2018a; Wu et al., 2019; Ding et al., 2021; Wu et al., 2022a), so we focused on the analysis of WRKY and HSF in this study. Notably, compared with the TKs treatment at non-HS, the members of the WRKY family that responded to TKs treatment increased by 50 WRKYs under HS (**Figures 8A, B** **)**. In 61 differentially-expressed-*WRKY* genes responding to TKs under HS, most *WRKYs* were down-regulated including 90% of *WRKY53* and all *WRKY2* (**Figure 9A**). Only eight *WRKY* genes were up-regulated under HS+TKs, of which *WRKY33* accounted for 37.5%. In the HSF family, 40 *HSFs* responded to HS+TKs treatment, of which 37 *HSFs* expression were up-regulated by TKs (**Figure 8D**). Moreover, we noted that the number of *HSFA2* transcripts accounts for 73% of the up-regulated *HSFs*. Interestingly, 14 *HSFs* down-regulated by TKs under non-HS were significantly up-regulated by HS+TKs. These results suggested that *HSFs*, especially *HSFA2*, may play a central role in heat tolerance induced by TKs.

Among the 50 most highly induced genes following HS+TKs treatment compared with HS treatment, 22% are *HSP70/90* genes (**Supplementary Table 2**). HSPs serve as molecular chaperones in the HSR, which are well-known targets of HSFs. 373 differentially-expressed-*HSP* genes were identified, and about 91% of the *HSP* genes were up-regulated by HS+TKs treatment compared with HS treatment, indicating that TKs treatment also had a strong promoting effect on *HSP* genes under HS conditions (**Figure 9B** **;** **Supplementary Table 3**).

The analysis of DEGs provided candidate genes, such as *HSFA2*, *WRKY33*, *HSP90*, and *HSP70* for further exploration of function in TKs-elicited thermotolerance. The above findings revealed that the mechanism of HS tolerance regulated by TKs in Lanzhou lily might be mainly involved in the HSF-HSP pathway.

### TKs treatment up-regulated heat-protective genes transcripts under HS

**Figure 10:**
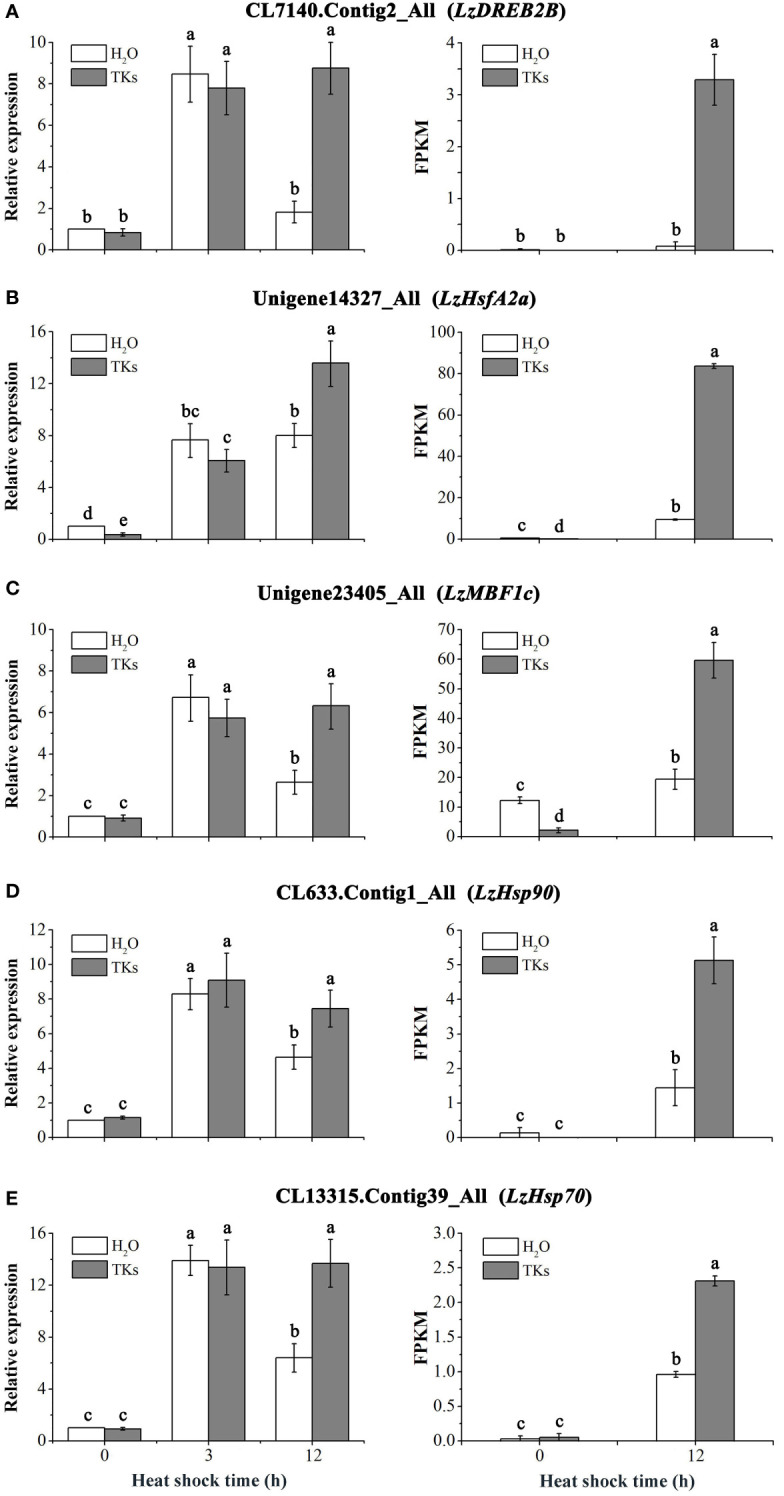
qRT-PCR analysis of HS-induced genes in Lanzhou lily leaves and validation of DEGs. Expression of *LzDREB2B* **(A)**, *LzHsfA2a* **(B)**, *LzMBF1c* **(C)**, *LzHsp90* **(D)**, and *LzHsp70* **(E)** in response to distilled water or 2 mg/L TKs treatment at 40°C heat treatment for different periods (0, 3, and 12 h). Samples treated with distilled water under normal conditions (22°C) were used for normalization, with their gene expression level set to 1. The relative expression level of each gene was calculated using the 2^–ΔΔCt^ method after normalizing for levels of *18S rRNA* in each sample. Each treatment included three plants. Data are means ± SD of three independent experiments. Different lowercase letters indicate significant differences at P <0.05 (Duncan test).

RNA-seq analysis revealed that the HSF-HSP pathway might mainly regulate the heat response networks in TKs-primed Lanzhou lily, so several heat-protective genes (*DREB*, *HSFA*, *HSP* and *MBF1c*) involved in this signal chain were selected for gene expression assay. The expression profiles of *DREB2B*, *HSFA2a*, *MBF1c*, *HSP90*, and *HSP70* genes in Lanzhou lily in response to HS and TKs treatment were quantified using qRT-PCR (**Figures 10A–E**). There was no significant difference in the expression level of these genes between TKs-treated and non-treated control plants under non-stressed conditions (22°C) except *LzHsfA2a* (**Figure 10B**). The expression of these genes in non-treated plants raised significantly after short-term (3 h) HS treatment, and then declined substantially after long-term (12 h) HS treatment with an exception of *LzHsfA2a* (**Figures 10A–E**). The results implied that *LzHsfA2a* might play an important role in the later stage of HSR in Lanzhou lily. TKs application had no influence on genes expression in short-term HS compared to non-treated control, but caused induction of those genes in long-term HS. The results indicated that TKs treatment could enhance the upregulation of heat-protective genes during long-term HS.

The five gene expression profiles of qRT-PCR in response to HS or HS+TKs treatment at 12 h were strongly consistent with those obtained from RNA-Seq (**Figures 10A–E**), suggesting that the data from the transcriptome were reliable.

### LzHsfA2a-1 shared high similarity to LlHsfA2 sequence

**Figure 11:**
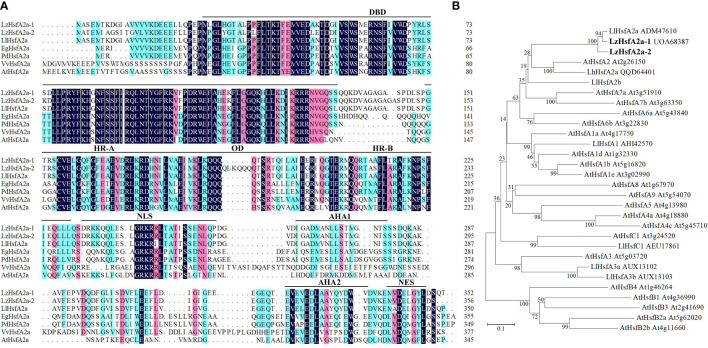
Sequences alignment and phylogenetic relationship of LzHsfA2a with its homologs from other species. **(A)** Alignment of the deduced amino acid sequences of LzHsfA2a-1, LzHsfA2a-2 and other plant HsfA2a. LzHsfA2a proteins contained conserved domains: DBD (DNA binding domain), OD (oligomerization domain), HR-A/B (hydrophobic amino acid residues), NLS (nuclear localization signal), AHA (aromatic, hydrophobic and acidic amino acid residues), and NES (nuclear export signal). **(B)** Phylogenetic relationship between LzHsfA2a-1, LzHsfA2a-2, and other plant HSF proteins. The phylogenetic tree was constructed by the neighbor joining method using MEGA 7.0 software. LzHsfA2a-1 and LzHsfA2a-2 are members of class HSFA2. Node values are bootstrap percentages based on 1000 replicates. The branch length scale bar indicates the evolutionary distance of 0.1 amino acid substitutions per site.

The gene expression profiles derived from RNA-seq and qRT-PCR revealed that *LzHsfA2a* in response to long-term HS with or without TKs treatment, which might contribute to the improved thermotolerance of Lanzhou lily treated with TKs, so *LzHsfA2a* was chosen as a functional candidate gene for further study. The *LzHsfA2a-1* (*LzHsfA2a*) and *LzHsfA2a-2* genes were isolated from Lanzhou lily, which contains a 1059-bp open reading frame (ORF) encoding a protein of 352 amino acid residues and a 1071-bp ORF encoding a protein of 356 amino acid residues, respectively (**Figure 11A**). Phylogenetic analysis was performed among LzHsfA2a-1 and LzHsfA2a-2, HSFs in lily and all the members of HSFs in *Arabidopsis* (**Figure 11B**). LzHsfA2a-1 (UOA68387) and LzHsfA2a-2 respectively showed 95.44% and 89.33% similarity to LlHsfA2a (ADM47610) which had properties of heat resistance in ornamental lily (Xin et al., 2010; Zhou Y, et al., 2022). Multiple alignments and sequence analysis revealed that the deduced amino acid sequence of LzHsfA2a-1 contained typical functional domains, including DNA binding domain (DBD), oligomerization domain (OD), hydrophobic heptad repeat A/B (HR-A/B), nuclear localization signal (NLS), aromatic, hydrophobic and acidic amino acid residues (AHA), and nuclear export signal (NES) (**Figure 11A**).

Because of the sustained high-level expression in HSR and high similarity to LlHsfA2a sequence, we speculate that LzHsfA2a-1 has the function of heat tolerance in Lanzhou lily. Moreover, the long-term HS-induced *LzHsfA2a-1* expression was up-regulated by TKs treatment, so LzHsfA2a-1 likely plays a crucial role in TKs-induced thermotolerance of Lanzhou lily.

## Discussion

### TKs from *Trichoderma longibrachiatum* SMF2 enhanced tolerance to HS on Lanzhou lily

HS is becoming one of the major limiting factors for food security due to global warming, so it is necessary to take effective measures to improve plant thermotolerance. Improving heat tolerance in crop plants through conventional breeding is a long and capital-intensive process, while genetic engineering is associated with ethical and social acceptance issues (Backer et al., 2018). Previous studies have suggested that application of plant protectants or inducers, such as osmoprotectants, PGRs, signaling molecules, mineral elements, amino acids, polypeptides, or plant growth-promoting microorganisms (PGPMs), could be another way to solve this problem (Hasanuzzaman et al., 2013; Backer et al., 2018). Melatonin pretreatment has been shown to improve heat stress tolerance of *Arabidopsis* and tall fescue (Shi et al., 2015; Alam et al., 2018), while the thermotolerance of maize and lily was enhanced by exogenous Ca^2+^ (Gong et al., 1997; Cao et al., 2013). Maize seedlings treated with exogenous trehalose show a higher survival percentage under HS (Li Z, et al., 2014). SA has been implicated in heat resistance in *Arabidopsis* (Larkindale and Knight, 2002; Clarke et al., 2004), grapevine (Wang et al., 2010), rice (Lu et al., 2009), pea (Pan et al., 2006), lily (Chen et al., 2008) and grape berry (Wen et al., 2008). Exogenous application of ABA and methyl jasmonate protects rice and *Arabidopsis* from HS injury, respectively (Clarke et al., 2009; Liu et al., 2022). Pretreatment with EBL in melon alleviates HT-caused growth suppression (Zhang et al., 2013), and the similar findings were observed in tomato and *Camellia sinensis* L. (Mazorra et al., 2002; Li et al., 2016). It has been reported that PGPMs and elicitors secreted from PGPMs have an important function in plant stress tolerance, including heat tolerance (Backer et al., 2018). *Pst* DC3000-induced defense response alleviates the injury caused by subsequent HS in *Arabidopsis* (Tuang et al., 2020). GAs produced by *Gibberella fujikuroi* was reported to promote plant growth attributes of date palms under HT stress (Khan et al., 2020). *Trichoderma* spp. are well known as important biological control agents of plant diseases (Howell, 2003). Trichokonins are peptaibols produced by *Trichoderma longibrachiatum* SMF2, consisting of two major isoforms, 20‐aa TKs (TKA) and 11‐aa TKs (TKB) (Xie et al., 2014). They were previously found to exhibit broad-spectrum antimicrobial activity against bacteria, fungal phytopathogens and TMV. Furthermore, they induced systemic resistance against *Botrytis cinerea* infection in moth orchid and *Pcc* infection in Chinese cabbage (Li H, et al., 2014; Zhao et al., 2018). However, there have been no studies on the function of TKs on plant abiotic stresses. This study showed that TKs treatment improved the thermotolerance of Lanzhou lily plants, as evidenced by a higher survival rate under HS (**Figure 1**). This is the first report that exogenous application of TKs can enhance plant resistance to HS. Given its advantages of being highly effective, eco-friendly, easily obtainable, and low-cost, TKs is expected to be a commercial plant inducer in the future.

### TKs treatment altered heat-resistance-associated physiology properties of Lanzhou lily to improve thermotolerance

Plants develop various defense strategies to buffer the damages imposed by HS, including morphological, physiological and biochemical responses. Leaf RWC is an important indicator of plant water status. In this study, leaf RWC showed a decreasing trend with increasing exposure time to HT, which was in agreement with results reported in hyacinth bean (Rai et al., 2015) and *Festuca arundinacea* (Xu et al., 2007). Our results revealed that TKs priming maintained a relatively higher leaf RWC under HS conditions (**Figure 2B**). Similar results were obtained by Li et al. (2014) who found that RWC was recovered by the application of NO during HS. HS-induced impairment of photosynthesis includes direct damage to the photosynthetic apparatus and inhibition of chlorophyll biosynthesis. A reduction in chlorophyll content and *P*_n_ as a result of HT was observed in peas (Haldimann and Feller, 2005), which was in conformity with our findings in this research. We found that TKs treatment prevented the decrease in chlorophyll content and *P*_n_ caused by HS (**Figures 2C, D** **)**, potentially resulting in enhanced thermotolerance in Lanzhou lily. The similar observation had also been made by Alam et al. (2018) in tall fescue by exogenous melatonin. ROS, such as superoxide radical (O_2_^–^), hydroxyl radical (OH), hydrogen peroxide (H_2_O_2_), and single oxygen (^1^O_2_), serve as signaling messengers required for plant HSR at low levels (Mittler, 2017). However, the injury mechanisms under HT involve the overproduction of ROS leading to lipid peroxidation and increased electrolyte leakage. Consistent with studies on tall fescue and *Lilium longiflorum* (Yin et al., 2008; Alam et al., 2018), increases in MDA content and REL were found in Lanzhou lily leaves after long-term HS (**Figures 2A**, **3A**). To mitigate and repair the damage initiated by ROS, plants have evolved ROS-scavenging mechanism containing enzymatic systems (SOD, CAT, POD, APX, and GR) and non-enzymatic systems (AsA and GSH). As reported in ornamental lily (*Lilium longiflorum*), the enzyme activities of SOD, POD, CAT, APX and GR were stimulated and the content of AsA and GSH maintained high levels during 10 h HS at 37°C and 42°C, which resulted in low O_2_^–^ and H_2_O_2_ concentrations (Yin et al., 2008). There is increasing evidence that exogenous protectants can alleviate membrane injury induced by HS through improving antioxidant defense capacity. Applying exogenous rosmarinic acid in tomatoes enhances the activities of APX, CAT, GR, and DHAR and modulates the redox status of GSH and AsA to mitigate the lipid peroxidation caused by heat-induced oxidative stress (Zhou Z, et al., 2022). Employing exogenous ABA in rice relieves cell injury, as shown by a lower accumulation of MDA and REL under HS (Liu et al., 2022). In the present investigation, TKs showed a parallel function and mechanism in regulating the tolerance of Lanzhou lily plants to HS. SOD, POD and CAT activity were further promoted by TKs treatment in Lanzhou lily plants subjected to HS (**Figures 3B–D**), contributing to the reduced MDA and REL accumulation. The results were in coincidence with findings in tea plants treated with AMHA (Yang et al., 2023). The phytohormones ABA, BRs, SA, JA, auxin (IAA), cytokinin (CK), and ethylene (ET) integrate HT stimuli and endogenous signals to regulate plant defensive response to HS (Li et al., 2021). Generally, ABA is recognized as a stress hormone in plants. HS elicites a rapid and transient increase in endogenous ABA levels that confer thermal tolerance by increasing ROS levels to enhance antioxidant capacity (Larkindale et al., 2005; Kaya et al., 2019). In this work, the HT-induced ABA content was further promoted by TKs treatment (**Figure 4A**), which was accordant to the elevated antioxidase activity. Many researches have also reported the role and mechanism of SA in protecting plants against heat-induced damage. SA application ameliorates HS-induced damage by the improvement on plant growth, antioxidants level and photosynthetic efficiency (Shah Jahan et al, 2019; Wassie et al., 2020). Although the positive role of JA in plant heat tolerance is well documented, the underlying mechanisms are not well understood. Unlike the response pattern of ABA to HS, SA and JA content displayed a sustained reduction during 48-72 HS (**Figures 4B, C** **)**. However, SA and JA levels were induced by TKs under HS conditions, suggesting SA and JA were also related to TKs-induced thermotolerance acquisition of Lanzhou lily. Accordingly, TKs treatment affected the synthesis and signal transduction of these above phytohormones under HS, and TKs improved the heat tolerance of Lanzhou lily possibly by promoting ABA synthesis and signal transduction (**Figure 7**). Interestingly, TKs treatment increased SOD, CAT, and POD activities, and SA and JA content at early stage (24 h) under non-HS conditions, which coincides with the discovery that TKs are known as elicitor for plant induced resistance (Luo et al., 2010; Li H, et al., 2014; Zhao et al., 2018).

### HSF-HSP pathway was the dominant thermal response pathway in TKs-primed Lanzhou lily and LzHsfA2a-1 probably played a central role in TKs-induced thermotolerance

At the molecular level, genes responsible for the expression of osmoprotectants, detoxifying enzymes, transporters, and regulatory proteins are induced for protection from HS (Hasanuzzaman et al., 2013). HS signal transduction networks in plant cells mainly involve HSF-HSP, Ca^2+^-CaM, ROS, and hormone pathways, of which HSF-HSP is considered as the fundamental and master signaling pathway (Saini et al., 2022; Zhang et al., 2022). HSPs are major functional proteins induced by HS that are divided into HSP100, HSP90, HSP70, HSP60, and sHSP based on their molecular weight. HSPs function as molecular chaperones to renature a variety of proteins denatured by HS, which are associated with acquired thermotolerance in *Arabidopsis*, rice, tomato, Lanzhou lily, and other plants (Katiyar-Agarwal et al., 2003; Su and Li, 2008; Mu et al., 2013; Zhuang et al., 2020). HSFs are the terminal components of the HS signal transduction chain that can recognize and bind to HSEs (5′-nGAAnnTTCn-3′) in promoters to regulate the expression of *HSP* and other heat-responsive genes. According to the peculiarities of their flexible linkers and oligomerization domain, plant HSFs are classified into three families, HSFA, B, and C (Guo et al., 2016). In *Arabidopsis*, HSFA1s are identified as the master regulators in the early phase of HSR, while HSFA2 serves as core regulators in the late phase of HSR (Schramm et al., 2006; Yoshida et al., 2011). Upon HS, the transcript levels of *HSFA2* and *A6* members become the dominant *HSFs* in wheat (Xue et al., 2014). The natural variations of HSFA2 enhance thermotolerance in grapevine (Liu et al., 2023). HS-responsive transcription factors DREB2A and DREB2C are identified as acting upstream of HSFA3 for the establishment of thermotolerance (Schramm et al., 2008; Chen et al., 2010), while *MBF1c* closely involved in plant HSR is activated by HSFA1b, HSFA2, and DREB2A (Sakuma et al., 2006; Ogawa et al., 2007; Bechtold et al., 2013; Qin et al., 2015; Liu et al., 2023). Plant-specific WRKY TFs also function in plant HSR. AtWRKY25, AtWRKY26, and AtWRKY33 positively regulate the cooperation between the ET-activated and HSPs-related signaling pathways that mediate responses to HS (Li et al., 2011). HSFA1, HSFA2a, HSFA2b, HSFA3a, HSFA3b, HSFA4, DREB2B, MYB305, WRKY22, WRKY39, MBF1c, NAC014, HB16, and ERF110 have been identified from ornamental lily, and all of these TFs are positively correlated with lily thermotolerance (Xin et al., 2010; Gong et al., 2014; Wu et al., 2018a; Wu et al., 2018b; Wu et al., 2019; Ding et al., 2021; Wang C, et al., 2022; Wang Y, et al., 2022; Wu et al., 2021; Wu et al., 2022a; Wu et al., 2022b; Xin et al., 2017; Zhou Y, et al., 2022; Wu et al., 2023). However, only heat-inducible *HSP16.45* has been reported in edible Lanzhou lily (Mu et al., 2013).

To elucidate the molecular mechanism of TKs enhancing the thermotolerance of Lanzhou lily, RNA-seq and data analysis were conducted in this study. GO and KEGG enrichment analyses revealed that TKs treatment at non-HS conditions might mainly affect the carbohydrate metabolism of Lanzhou lily plants, while TKs treatment at HS conditions might improve the heat tolerance by reducing protein folding and enhancing cellular repair function (**Figures 5**, **6**). The analysis of DEGs revealed that the mechanism of HS tolerance regulated by TKs in Lanzhou lily might be mainly involved in the HSF-HSP pathway, in which *HSFA2* might be a central regulator (**Figures 8**, **9**). The expression patterns of *LzDREB2B*, *LzHsfA2a*, *LzMBF1c*, *LzHsp90*, and *LzHsp70* genes involved in HSF-HSP pathway in response to HS and TKs treatment were studied using qRT-PCR (**Figure 10**). The results indicated that the thermal-induced *LzDREB2B*, *LzHsfA2a*, *LzMBF1c*, *LzHsp90*, and *LzHsp70* transcripts were up-regulated by TKs treatment after long-term (12 h) HS, which validated the accuracy of the RNA-Seq data and was probably responsible for the improved thermotolerance of Lanzhou lily. We also discovered that *LzHsfA2a* activity positively and lastingly responded to HS, so *LzHsfA2a* was chosen as potential functional gene for further study. Subsequently, *LzHsfA2a-1* and *LzHsfA2a-2* genes were isolated from Lanzhou lily (**Figure 11**), and *LzHsfA2a-1* (*LzHsfA2a*) encodes a protein of 352 amino acid residues that shows 95.44% similarity to LlHsfA2a which had properties of heat resistance in *Lilium longiflorum* (Xin et al., 2010; Zhou Y, et al., 2022). As the result of the lasting response to HS, the elevated response to HS coupled with TKs treatment, and the high similarity to LlHsfA2a sequence, it can be speculated that LzHsfA2a-1 probably plays a vital role in regulation of TKs-induced thermotolerance of Lanzhou lily. Future studies to confirm the molecular function of *LzHsfA2a-1* and other candidate genes, such as *LzWRKY33*, and *LzHSPs*, will provide new data for understanding the TKs-regulated mechanism of HSR in Lanzhou lily.

## Conclusion

In summary, TKs application enhanced thermotolerance in Lanzhou lily plants *via* promoting photosynthetic capacity, water retention ability, antioxidant enzyme system activity, HS-related phytohormones level, and HS-protective genes transcription regulation. Therefore, this study not only provides a direct link between TKs and plant thermotolerance, but also supplies a promising strategy that can alleviate plant injury resulted from HS.

## Data availability statement

The original contributions presented in the study are publicly available. This data can be found here https://www.ncbi.nlm.nih.gov/bioproject/PRJNA934416.

## Author contributions

XC and JL contributed to the conceptualization of the study and writing of the original draft. XC, JS, DH, JL and ZW contributed to the methodology. HL, WY and TL performed data curation and graphing. DH contributed to the material preparation. XC, JS, HL, WY, TL, DH, JL and ZW contributed to the writing, reviewing, and editing. JL supervised the work. XC and JL contributed to funding acquisition. All the authors contributed to the manuscript revision and approved the submitted version.
