# Supplementary material for: Enhancing heat stress tolerance in Lanzhou lily (Lilium davidii var. unicolor) with Trichokonins isolated from Trichoderma longibrachiatum SMF2
Source: Front Plant Sci. 2023 Jun 7;14:1182977. doi: 10.3389/fpls.2023.1182977 (PMC10282843; doi:10.3389/fpls.2023.1182977)
Supplement: Supplementary file 1 [file DataSheet_1.docx]

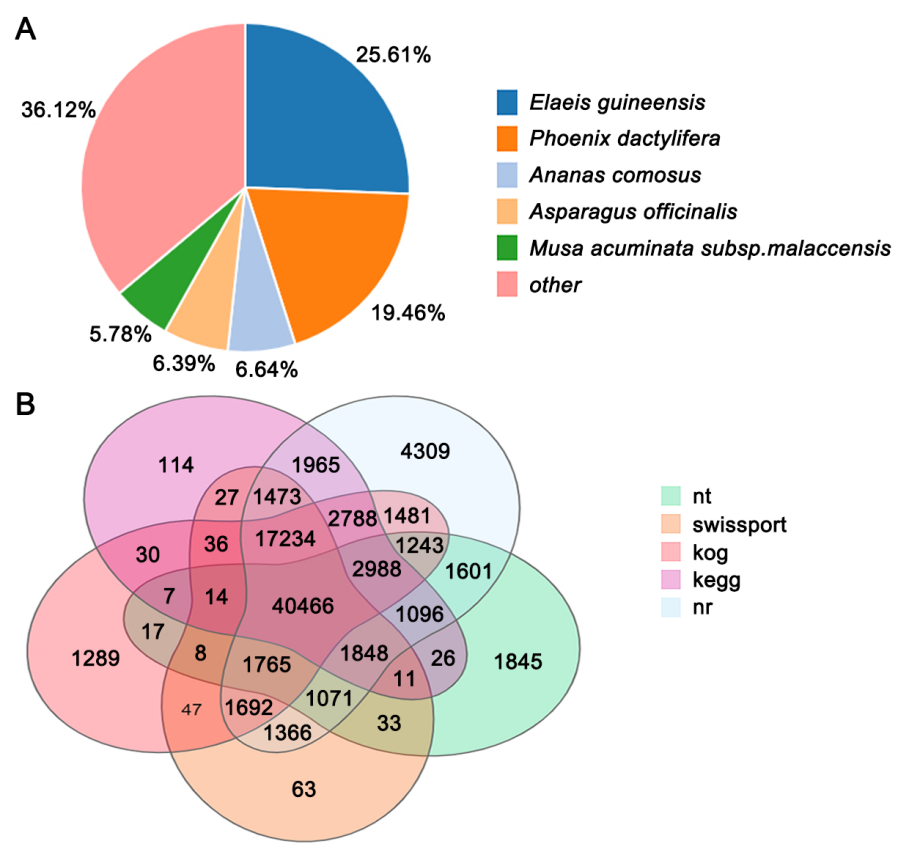


**Supplementary** **Figure 1. Comprehensive overview of de novo assembly of Lanzhou lily transcriptome.** **(A)** Species distribution of the top BLAST hits for Lanzhou lily assembled transcriptome. **(B)** Comprehensive overview of BLASTx alignment against the public protein databases including Nr, KEGG, COG, Swiss-Prot and InterPro proteins.
