# Supplementary material for: Enhancing heat stress tolerance in Lanzhou lily (Lilium davidii var. unicolor) with Trichokonins isolated from Trichoderma longibrachiatum SMF2
Source: Front Plant Sci. 2023 Jun 7;14:1182977. doi: 10.3389/fpls.2023.1182977 (PMC10282843; doi:10.3389/fpls.2023.1182977)
Supplement: Supplementary file 3 [file DataSheet_3.docx]

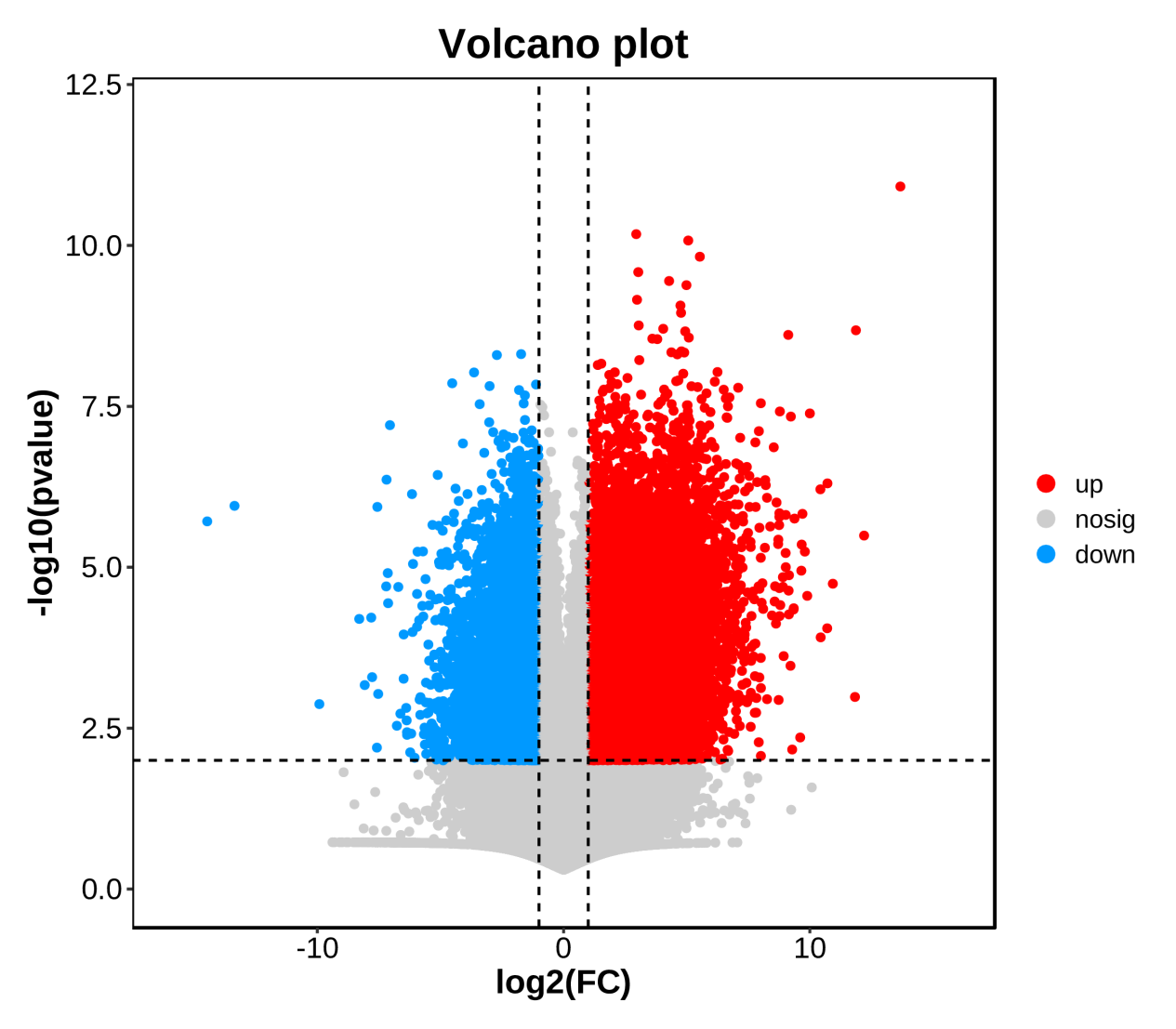


**Supplementary** **Figure 3. The volcano plot shows genes that were up-regulated, down-regulated, and no significantly changed at HS vs. HS+TKs.**
