# Supplementary material for: Enhancing heat stress tolerance in Lanzhou lily (Lilium davidii var. unicolor) with Trichokonins isolated from Trichoderma longibrachiatum SMF2
Source: Front Plant Sci. 2023 Jun 7;14:1182977. doi: 10.3389/fpls.2023.1182977 (PMC10282843; doi:10.3389/fpls.2023.1182977)
Supplement: Supplementary file 4 [file DataSheet_4.docx]

**Supplementary Table 1. Overview of the RNA-seq read data.**

| **Sample** | **Raw Reads**  **(M)** | **Clean Reads**  **(M)** | **Clean Reads Q20(%)** | **Clean Reads Q30(%)** |
| --- | --- | --- | --- | --- |
| W_1 | 49.15 | 43.53 | 96.16 | 87.05 |
| W_2 | 49.19 | 43.6 | 96.31 | 87.47 |
| W_3 | 49.19 | 43.41 | 96.2 | 87.15 |
| TKs_1 | 47.44 | 42.68 | 95.81 | 86.44 |
| TKs_2 | 49.19 | 44.1 | 96.07 | 87.15 |
| TKs_3 | 49.19 | 43.81 | 96.19 | 87.11 |
| HS_1 | 49.19 | 43.21 | 95.59 | 86.08 |
| HS_2 | 45.87 | 40.85 | 95.96 | 86.64 |
| HS_3 | 52.71 | 46.45 | 95.96 | 86.62 |
| HS+TKs_1 | 49.19 | 43.88 | 95.72 | 86.23 |
| HS+TKs_2 | 49.19 | 43.67 | 96.25 | 87.31 |
| HS+TKs_3 | 49.19 | 43.59 | 96.07 | 86.88 |
